# The Quality of Discharge Summaries at Al-Shaab Hospital, Sudan, in 2022: The First Cycle of a Clinical Audit

**DOI:** 10.7759/cureus.41620

**Published:** 2023-07-10

**Authors:** Abdullatif Yasir H Eissa, Ahmed Zaki W Mohamed Elhassan, Almothanna Zaki H Ahmed, Ammar Elgadi, Gaffar Alemam A Manhal, Mohamed H Fadul, Mohamed Ishag Ahmed, Abdalla Fadul, Islah Ismail Mekki

**Affiliations:** 1 Faculty of Medicine, University of Khartoum, Khartoum, SDN; 2 Department of Internal Medicine, Hamad Medical Corporation, Doha, QAT; 3 Department of Respiratory Medicine, Al-Shaab Teaching Hospital, Khartoum, SDN

**Keywords:** sudan, discharge interview, discharge counseling, hospital discharge, discharge summary, patient discharge, quality improvement, clinical audit

## Abstract

Background

The discharge summary is a vital component of the modern health system. It is defined as a synopsis of information regarding events occurring during the inpatient care of a patient, to allow for a safe, quick, and effective patient-centered discharge process. It contains important information about the patient’s hospital stay, including the reason for admission, treatment received, and follow-up needed. Low-quality discharge summaries pose a great risk to patient healthcare since the most frequent reason for error in clinical settings is poor communication. In the United Kingdom, the Professional Record Standards Body (PRSB) has adopted the Academy of Medical Royal Colleges (AoMRC) “Standards for the Clinical Structure and Content of Patient Records” and produced a standard discharge summary form. This study aimed to assess the quality of discharge summaries at Al-Shaab Hospital in Sudan in terms of information, filling adequacy, and adherence to international guidelines and evaluate the discharge interviews.

Methods

A cross-sectional institution-based study was conducted in the period of September to December 2022 at Al-Shaab Teaching Hospital in Khartoum, Sudan. Systematic random sampling was used to select the study participants from the discharged patients. A total of 70 patients were met in their wards over a period of two months, and the contents of their discharge cards were compared to items on an online checklist based on the Professional Record Standards Body (PRSB) and the Academy of Medical Royal Colleges (AoMRC) standard discharge summary. The patients were also interviewed to assess their knowledge regarding their discharge information.

Results

The hospital’s discharge summary form contained only four headings: date, patient name, age, and ID number. The assessed cards were found to be missing valuable information, including date of admission (missing in 83%), filling doctor’s name (missing in 71%), and medication changes (missing in 70%). Only half of the summaries were clearly readable. The majority of patients had poor knowledge regarding their medication side effects (89%) and how to act in an emergency (86%), while knowledge of medication doses and follow-up details was good in 80% and 66%, respectively.

Conclusion

The patients are discharged with inadequately filled discharge forms. This may be due to the poor design of the form, so a newly designed form will be proposed, based on international standards. The discharge interview is also in need of improvement, to make sure patients are fully aware of their condition.

## Introduction

Clinical audit is one of the effective ways of quality improvement in the healthcare system. It is believed that audits lead to improved patient care and better system and administration [1].

The discharge summary is a vital component of the modern health system. One study defined a hospital discharge summary as a synopsis of information that outlines all the events occurring during the hospital stay of the patient [2]. It contains important information about the patient’s hospital visit, including the reason for admission, the results of any tests, the treatment received, any changes to medication, and what follow-up is needed. Ideally, there should be an electronic version of the discharge summary, stored in the hospital’s database, and a paper copy given to the patients at discharge. In addition, each general practitioner (GP) should have access to their patients’ discharge summaries [3].

The summary is the main method for patient information transfer between different healthcare providers and healthcare levels [4]. In one study, it was found that most physicians believe it is a good idea to give patients a card containing information about their medications and what steps they need to take next [3]. The discharge card is thought to be the most effective way to deliver such information to the patient. Patients also assured that the summary helps them understand how to manage their disease once they leave the hospital and what steps they should take after discharge [5].

The quality of the information supplied in discharge summaries is crucial for a safe, quick, and effective patient-centered discharge process [6]. Low-quality discharge summaries pose a great risk to patient healthcare, since the most frequent reason for error in clinical settings, according to the Institute of Medicine, is poor communication [7]. For many patients, the time after their release from hospital is a time of vulnerability. Missing important information or sharing erroneous information might lead to unintentional omission or delay of necessary treatment, as well as task duplication [6]. The detrimental impact on healthcare safety and quality is a key concern.

Having a standard template for a discharge form is recommended, as it was found that this reduces the chance of missing details [8]. It also ensures consistency and reduces chances of human error, as well as ensuring that the same quality of discharge is given to each and every patient, in each and every hospital. It is recognized that a discharge report ultimately enables doctors to give patients the best care possible and ensures a high level of patient safety is maintained [6]. In the United Kingdom, the Professional Record Standards Body (PRSB) has adopted the Academy of Medical Royal Colleges (AoMRC) “Standards for the Clinical Structure and Content of Patient Records” and produced guidelines on the discharge summary, which should include the following: patient’s details (name, ID number, date of birth, address, and individual requirements), GP details (name and address), admission details (date, source, method, and reason), clinical narrative (summary of the encounter), procedures and operations, investigations and results, medication changes and discharge medications details, allergies, risks and warnings, diagnoses at discharge, discharge details (date, consultant, department, location, and destination), advice, recommendations and future plans (follow-up details), legal information and capacity, patient and carer concerns, wishes and expectations, and the filling doctor’s name, grade/designation, and signature [9,10].

The discharge interview or counseling is the process in which the doctor communicates the discharge information to the patient or co-patient. The doctor must deliver all important information to the patient regarding the patient’s diagnosis, medications and how to use them, prognosis, how to act in an emergency, special instructions (if any), and others. The doctor should deliver the information in a way that leaves the patient satisfied with the interview. Answering the patient’s questions and responding to their concerns are vital components of a successful discharge interview.

In Sudan, the situation is far from ideal, as discharge summaries may not even be issued for all patients. Furthermore, the quality of those issued is questionable. There is a lack of knowledge among patients about their disease, its consequences, and treatment due to the inadequacy of the information provided in their discharge summaries.

Al-Shaab Teaching Hospital is located in Khartoum, the capital of Sudan. It offers emergency, inpatient, and outpatient care for cardiology, pulmonology, and cardiothoracic surgery cases. It has the first cardiac catheterization laboratory in Sudan, which was established in 1974 [11].

In Al-Shaab Hospital, patients are discharged from three hospital sites: the wards, the catheterization laboratory, and the emergency room. The call for discharge is done mainly during the daily rounds by the consultant or the senior registrar in the consultant’s team. Unfortunately, these rounds do not have a fixed starting time. The discharge summary is filled out by any doctor in the team and then handed to the patient later in the day before they leave the hospital.

This audit, which is, as far as we know, the first to be done in Al-Shaab Teaching Hospital, will help improve the quality of discharge summaries and interviews and the whole healthcare process for the benefit of patients and the healthcare system.

## Materials and methods

A cross-sectional institution-based study was conducted from September to December 2022 at Al-Shaab Teaching Hospital in Khartoum, Sudan, to evaluate the discharge summary and discharge interview. The study was the first cycle of a clinical audit. The guidelines on standard discharge summaries developed by the PRSB and AoMRC were used as a reference by the authors.

The sample size was calculated using Epi Info statistical software (Centers for Disease Control and Prevention, Atlanta, GA, USA). Population (N) was estimated as 450 (total discharges in two months, based on the hospital records), the confidence interval (CI) was set to 95%, and the margin of error to 10%. The calculated sample size (n) was 80 participants.

Data were collected in two phases. The first phase consisted of obtaining a blank (unfilled) copy of the hospital’s discharge card. The card was analyzed in terms of size, headings present, and space available for writing. The second phase of data collection was conducted during the months of October and November 2022 (a total of eight weeks). The hospital has seven wards, two of which were temporarily closed for maintenance. Data were collected from the five wards as well as the catheterization laboratory. Discharges were made from Sunday to Thursday every week. On each day, a list of the patients set for discharge was made and used as a sampling frame. The discharge rate was variable through the different days and weeks of the data collection period. The number of discharged patients ranged from six to 12 per day, and not all wards had patients for discharge every day. Systematic random sampling was used to select the study participants from the sampling frame, using a random number generator and a sampling interval of 6. This sampling interval was obtained by dividing the number of the estimated population (N=450) by the sample size (n=80). Consequently, one or two patients were randomly selected from the 6-12 discharged patients each day. The participants were then contacted in their wards to evaluate their discharge summaries using an online checklist and to assess their knowledge about their discharge information. Patients who refused to participate were excluded. Because the working shifts in the hospital were distributed by days, all discharging doctors were covered during the data collection period. By the end of the data collection period, there were 437 discharges and 70 randomly selected participants, achieving 88% of the calculated sample size.

The cards were evaluated using an online checklist developed by the authors for the presence of the following items: patient’s name, patient’s ID number, patient’s date of birth or age, date of discharge, diagnosis at discharge, consultant’s name, date and source of admission, the reason for admission, clinical narrative, procedures done during the hospital stay, investigations and results, treatment received in hospital, medication changes, discharge medications and medication recommendations, follow-up details, and the filling doctor’s name, grade/designation, and signature.

The second phase also comprised an interview with the patient, to explore their knowledge regarding their discharge and their condition in general. Patients were asked about the following domains: understanding of diagnosis, discharge medications and their doses, discharge medications’ potential side effects, follow-up details, understanding of how to act in an emergency, and special instructions from the doctor. Patients’ responses to each domain were classified as either “good” or “poor.” Lastly, the handwriting was evaluated and classified as clearly readable, readable with effort, or unreadable.

Data were collected using online Google Forms consisting of a checklist of the aforementioned items, filled out by the data collectors. Data were analyzed using Microsoft Office Excel (Microsoft Corp., Redmond, WA, USA). Descriptive statistics such as percentages and frequencies describing the filling and completeness of the discharge summary information and patients’ perception of the discharge information were used, and the results were illustrated in figures.

Ethical approval was obtained from the Research and Training Administration at Al-Shaab Teaching Hospital. Informed consent was taken from each participant.

## Results

The discharge summary form of Al-Shaab Hospital contained only four headings: date, patient name, age, and ID number. The remainder of the form (front and back) was blank to be filled by the doctor. The form measured 23 cm × 15.7 cm. A sample of the standard discharge summary form used currently at Al-Shaab Hospital is shown in the Appendices section.

**Figure 1:**
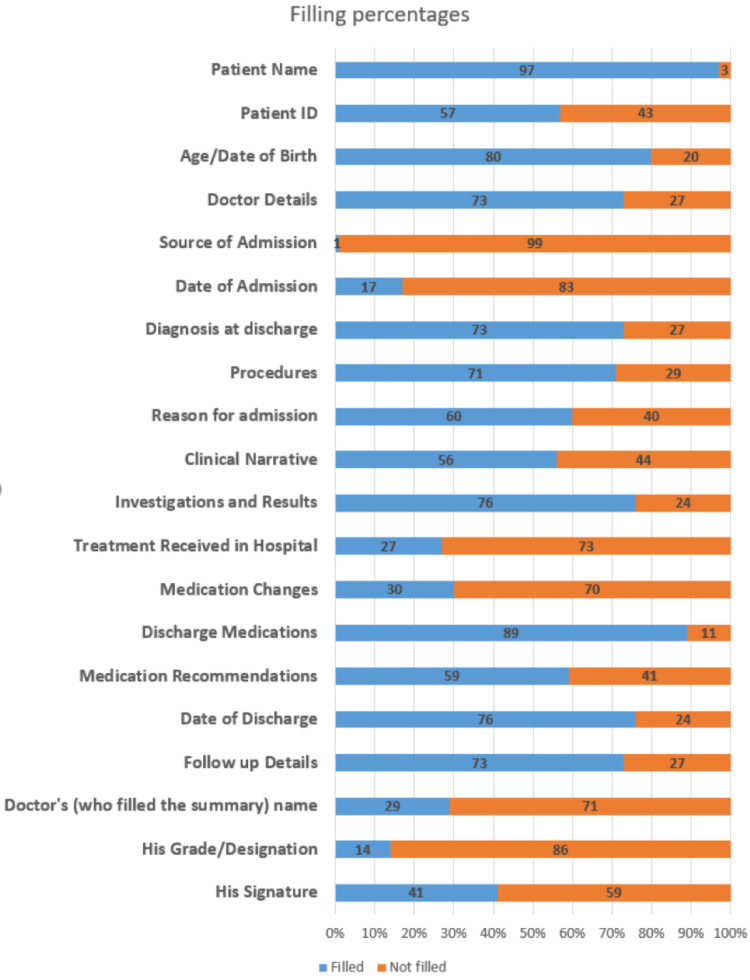
Percentages of filling of the discharge summary information at Al-Shaab Hospital (n=70).

The assessed cards were found to be missing valuable information, including date of admission (missing in 83%), filling doctor’s name (missing in 71%), and medication changes (missing in 70%). The complete details of the filling are illustrated in Figure 1.

None of the summaries contained any information about the following items (i.e., they were filled in 0% of the summaries): patient sex, address, and contact, discharge destination, allergies, risks and warnings, legal information and capacity, and patient and carer concerns, wishes, and expectations.

**Figure 2:**
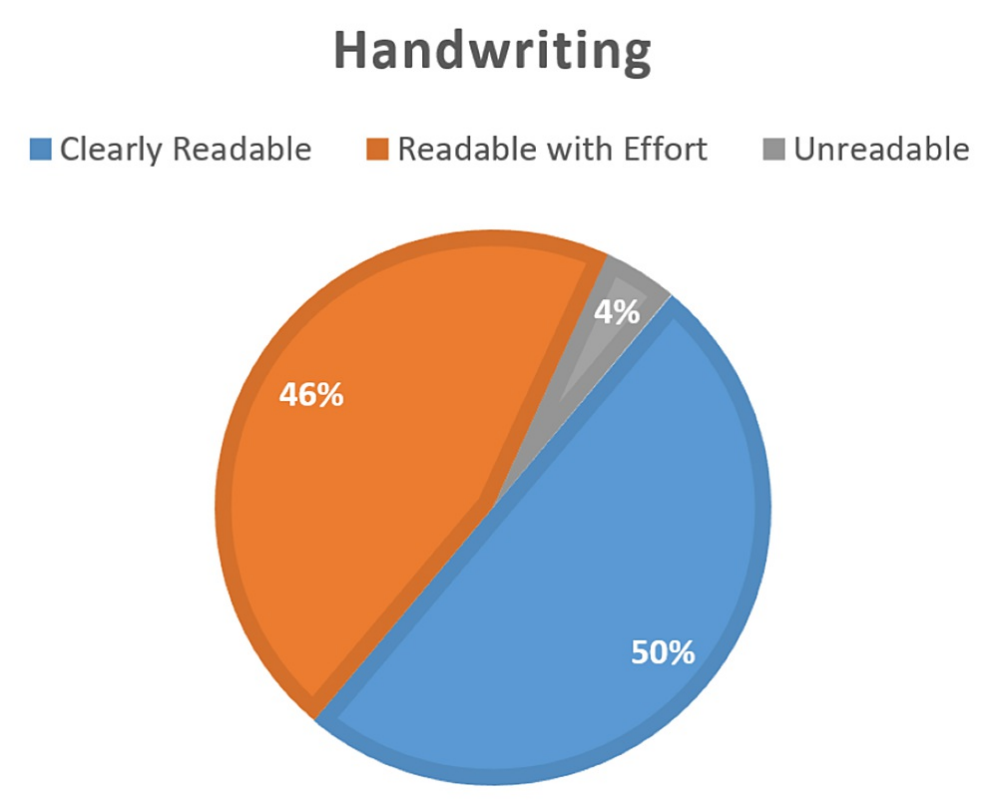
Handwriting of the discharge summaries at Al-Shaab Hospital (n=70).

The handwriting of half of the summaries was clear and readable (50%, n=35). Only a few had unreadable handwriting (4%, n=3) (Figure 2).

**Figure 3:**
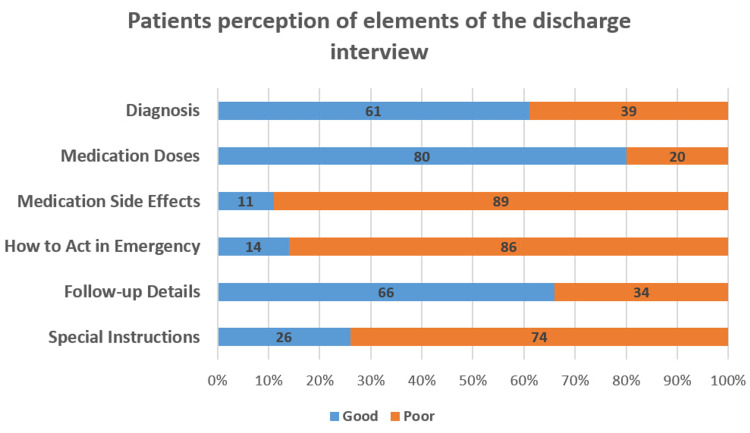
Patients’ perception of the elements of the discharge interview at Al-Shaab Hospital (n=70).

Of the patients, 89% (n= 62) did not know about the potential side effects of their medications. On the other hand, most of the patients showed a good understanding of their medication doses (80%, n=56) and their follow-up details (66%, n=46) (Figure 3).

## Discussion

Of the discharge summaries included in the study, 70% did not address medication changes during the patient’s admission. Medication changes during admission were considered by Scarfield et al. [12] as one of the 10 core items to be included in a discharge summary, based on inputs from bodies such as the Royal College of Physicians and the Professional Record Standards Body. The inclusion of such an item is important to keep the patient’s general practitioner (GP) in the picture regarding changes to previously prescribed medicines. Since the GP is often skipped in the Sudanese population’s pattern of treatment-seeking behavior, and patients jump to seeking tertiary/specialist level care, perhaps this part of the summary is deemed less important by physicians.

Only 4% of the assessed discharge cards were written in handwriting considered to be “unreadable” by the authors. Although discharge summaries are intended as a means of communication between physicians, some doctors believe that cards should be written in a manner that is also easily understood by patients [4]. Despite the handwriting being readable on the majority of the summaries, the cards were written in English, meaning the vast majority of the patients at the studied hospital will not be able to decipher the contents of the discharge card as they can only read their mother language, Arabic. This is due to the hospital’s role as one of Sudan’s biggest public hospitals, which accommodates patients from many rural areas of the country, with lower socioeconomic status and levels of education. This signifies the importance of adequate discharge interviews in the study setting.

One of the more surprising findings of the study was that only 11% of the patients were told about their prescribed medications’ side effects. The Adult Inpatient Survey done in the UK in 2020 revealed that less than a third of the respondents received an explanation from a member of staff about medication side effects to watch for when they went home from the hospital [13]. The urgent and emergency care survey, done in the same year, revealed that 60% of the participants received an explanation of some sort regarding their medications’ side effect profile. The extent to which patients should be told about potential side effects of treatment is a debated issue [14], especially while taking nocebo effects into consideration. A systematic review of surveys assessing patient attitudes toward side effect information showed that the majority of patients wanted to be fully aware of possible side effects, while only a small minority wanted little or no information at all [15]. The reasons why doctors refrain from explaining side effect profiles to their patients in the study setting is an area for further research.

Limitations of the study included a lack of similar studies, especially in the region, to aid in designing the study methodology and comparing the findings. Also, the study included only one hospital, which may limit the generalizability of the results to other institutions, although this is not the main objective of the audit.

## Conclusions

The discharge summary form at Al-Shaab Hospital is poorly designed and inadequately filled, and only half of all issued forms are clearly readable. In addition, discharged patients lack knowledge regarding crucial aspects of their condition.

Modifications to be made to the hospital’s discharge system include designing a new guideline-adherent form, training the doctors about the discharge summary and interview, and educating patients about the importance of the information they are given when discharged. These recommendations will be implemented in the second cycle of this clinical audit through workshops and wall posters. A new discharge summary form will also be designed in accordance with the PRSB and AoMRC guidelines (Appendices). Afterward, the quality of discharge summaries will be re-audited.

## Figures and Tables

**Figure 4:**
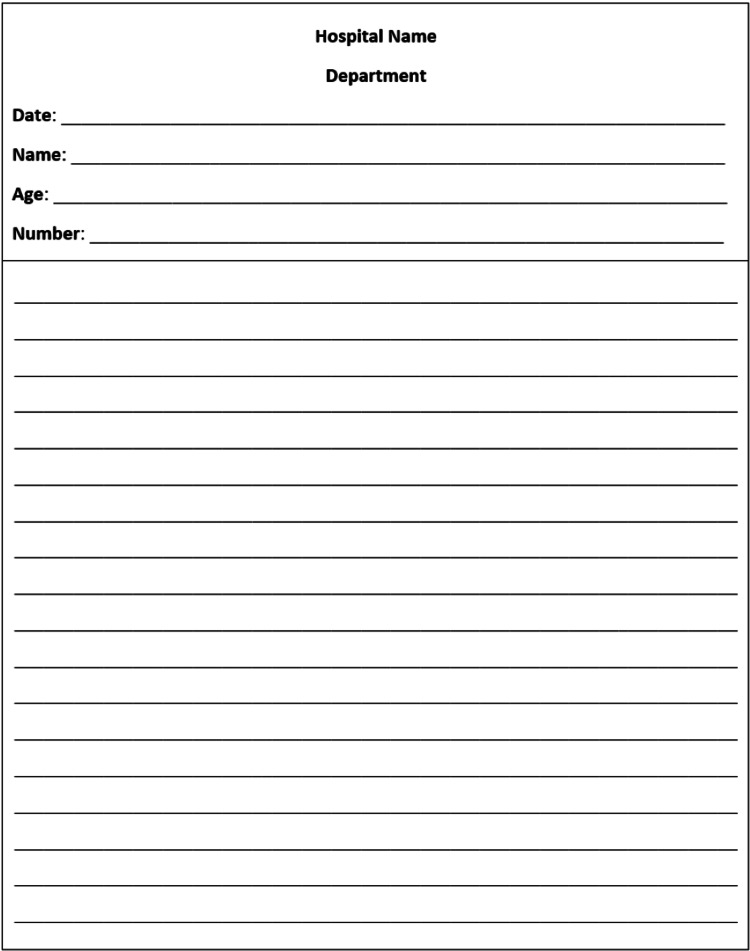
Current discharge summary form used at Al-Shaab Hospital.

**Figure 5:**
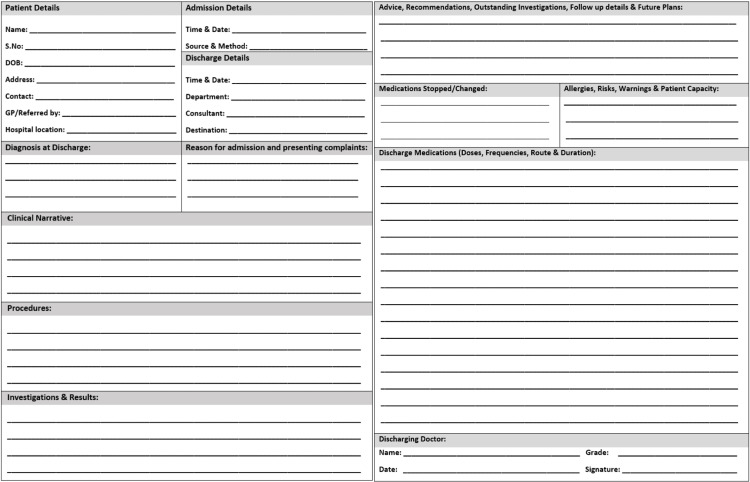
New discharge summary form proposed by the authors to be implemented at Al-Shaab Hospital: front (left) and back (right).

## Appendices

Figure 4 shows a sample of the standard discharge summary form used currently at Al-Shaab Hospital.

Figure 5 shows the new discharge summary form designed according to the PRSB and AoMRC guidelines.
